# Modeling Design Iteration in Product Design and Development and Its Solution by a Novel Artificial Bee Colony Algorithm

**DOI:** 10.1155/2014/240828

**Published:** 2014-11-06

**Authors:** Tinggui Chen, Renbin Xiao

**Affiliations:** ^1^College of Computer Science & Information Engineering, Zhejiang Gongshang University, Hangzhou, Zhejiang 310018, China; ^2^Institute of Systems Engineering, Huazhong University of Science and Technology, Wuhan, Hubei 430074, China

## Abstract

Due to fierce market competition, how to improve product quality and reduce development cost determines the core competitiveness of enterprises. However, design iteration generally causes increases of product cost and delays of development time as well, so how to identify and model couplings among tasks in product design and development has become an important issue for enterprises to settle. In this paper, the shortcomings existing in WTM model are discussed and tearing approach as well as inner iteration method is used to complement the classic WTM model. In addition, the ABC algorithm is also introduced to find out the optimal decoupling schemes. In this paper, firstly, tearing approach and inner iteration method are analyzed for solving coupled sets. Secondly, a hybrid iteration model combining these two technologies is set up. Thirdly, a high-performance swarm intelligence algorithm, artificial bee colony, is adopted to realize problem-solving. Finally, an engineering design of a chemical processing system is given in order to verify its reasonability and effectiveness.

## 1. Introduction

Due to fierce market competition, product design and development process is faced with a huge challenge. In addition, in the initial stage of industrialization, competitiveness mainly lies with the prices of products. Only if the products were cheap and usable, would they be of competitive advantage in the market. This type of competition is named the cost-based competition. However, with the development of economy, the quality, time-to-market, and service turned up trumps, which led to the competition being quality based as well as time based. As a result, to succeed in this type of competition, it is necessary for most of enterprises to introduce some new competitive products more quickly so as to occupy the global market share. It also means that new product development has become a key factor to keep the core competitiveness. Therefore, many enterprises adopt concurrent engineering (CE) technology to support product design and development. Nevertheless, due to the existing of coupling in product design and development, it is difficult to manage this process. Particularly when take execution may produce new information flow or affect other interdependent tasks, more complex information flows among interdependent tasks will be generated. At the same time, due to the randomness of information flow, incomplete information may often be used for design decision, which usually leads to design iteration [1]. Design iteration generally causes increases of product cost and delays of development time as well, so how to identify and model couplings among tasks in product design and development has become an important issue for enterprises to settle.

Many of the traditional project management techniques (e.g., Gantt chart, critical path method (CPM), and program evaluation and review (PERT)) only describe the sequential and parallel relationships, not the interdependent relationships in tasks. The design structure matrix (DSM) model presented by Steward [2] can express the interdependent relationships as well as the iterations induced by the relationships. It is a useful tool in concurrent engineering management and implementation. Moreover, in practical product development process, resource constraints from machine equipment, staffs, and so on should be considered, but the traditional methods cannot deal with this problem. Therefore, in this paper, we use DSM to identify and analyze design iteration. In current researches, only valid iterations were considered, but some invalid especially harmful ones were not studied. However, due to the existing of these invalid iterations, the whole product design and development process may not be convergent. As a result, how to avoid these harmful iterations needs further study.

In this paper, we use tearing approach combined with inner iteration technology to deal with task couplings, in which tearing approach is used to decompose a large coupling set into some small ones and the inner iteration technology to find out iteration cost. The paper is organized as follows. In Section 2, we survey the previous literatures on disposal of coupled relationships.  Section 3 presents the model for solving coupled task sets based on tearing approach and inner iteration technology. In Section 4, an efficient artificial bee colony algorithm (ABC) is used to search for a near-optimal solution of the model. In Section 5, the model is applied to an engineering design of a chemical processing system and some discussion on the obtained results is also given.  Section 6 offers our concluding remarks and potential extensions of this research.

## 2. Related Works

DSM is an efficient management tool for new product development. In the past decades, many researches have shown its efficiency. Currently, DSM has been widely used in decomposition and clustering of large-scale projects [3, 4], identification of task couplings and minimization of project durations [5, 6], project scheduling [7–10], and so on. Because coupling of tasks is a key characteristic of product development, how to deal with couplings among tasks is a hot issue in present.

Yan et al. [11, 12] focused upon the optimization of the concurrency between upstream product design task and downstream process design tasks in the concurrent engineering product development pattern. First, a new model of concurrent product development process, that is, the design task group model, was built. In this model, the product and process design tasks were carried out concurrently with the whole design process divided into several stages, every two of which are separated by a design review task. The design review tasks might lead to design iterations at a certain rate of probability. Therefore, a probability theory-based method was proposed to compute the mean duration of the design task group and the mean workloads of all the design and review tasks, with design iterations taken into consideration. Then, the problem of concurrency optimization was defined mathematically, whose objective was to minimize the total costs for delay of design task group completion time and unnecessary design revision workloads. Their research proved that the cost function was convex with respect to the concurrent (or overlap) degree between design tasks and that it must have a minimum value at a unique optimum point.

Huang and Gu [13, 14] viewed the product development process as a dynamic system with feedback on the basis of feedback control theory. The dynamic model and its design structure matrix were developed. The model and its design structure matrix could be divided farther to reflect the interaction and feedback of design information. The mode and direction of the development process could be selected to satisfy constraints of process data flow and process control. A fuzzy evaluation method was presented to evaluate the performance of the dynamic development process; this allowed the development process to be optimized based on reorganizing design constraints, reorganizing design processes, and reorganizing designer's preferences. Finally, an application shows that modeling the product development process as a dynamic system with feedback was a very effective method for realizing life cycle design, optimizing the whole development process, improving the degree of concurrent, speeding information flow, and reducing modification frequency. However, due to complexity of product development, this model did not consider the currency and overlapping among tasks. Its efficiency needs further study and verification.

Zhang et al. [15] constructed a new method to measure the coupled strength and to calculate the first iteration's gross workload of a different sequence of coupled tasks, thereby ascertaining the best sequence of coupled tasks based on existent research. However, this model may not correspond to real-world product development process and it is also dependent on expert's experiences. Moreover, Xiao et al. [16] adopted analytic hierarchy process (AHP) to deal with coupling tasks, which might cause quality loss.

Smith and Eppinger [17, 18] set up two different iteration models based on DSM. One is the sequential iteration model and the other is the parallel iteration model. The former supposed that coupled tasks were executed one after the other and rework was governed by a probabilistic rule. Repetition probabilities and task durations were assumed constant in time. The process was modeled as a Markov chain and the analysis could be used to compute lead time for purely sequential case and to identify an optimal sequence of the coupled tasks to minimize iteration time. The main limitation of this model is that how to determine repetition and rework probabilities is difficult. The latter supposed that the coupled design tasks were all executed in parallel and iteration was governed by a linear rework rule. This model used extended DSM called work transformation matrix (WTM) to identify the iteration drivers and the nature and rate of convergence of the process. WTM has been popularly used in many areas. For instance, Fontanella et al. [19] developed a systematic representation of the work transformation matrix method, with a discrete state-space description of the development process. With this representation, the dynamics of the development process can be easily investigated and predicted, using well-established discrete system analysis and control synthesis techniques. In addition, Ong et al. [20] developed nonhomogenous and homogenous state-space concepts, where the nonhomogenous one monitored and controlled the stability and the convergence rate of development tasks and at the same time predicted the number of development iterations; the homogenous one did not consider external disturbances and its response was only due to initial conditions.

Xiao et al. [21] put forward a model for solving coupled task sets based on resource leveling strategy. However, it is hypothesized that once resources allocated to coupled task sets are ascertained, then, in all iterations' process, they no longer change. It does not exactly accord with the real product development process. So, the authors [22] further proposed an approach to analyze development iteration based on feedback control theory in a dynamic environment. Firstly, the uncertain factors, such as task durations, output branches of tasks, and resource allocations, existing in product development were discussed. Secondly, a satisfaction degree-based feedback control approach is put forward. This approach includes two scenarios: identifying of a satisfaction degree and monitoring and controlling of iteration process. In the end, an example of a crane development was provided to illustrate the analysis and disposing process.

Different from the above research, we propose a method to solve coupled task sets combined with tearing approach and inner iteration technology in this paper. Its obvious advantages lie in identifying invalid iteration process and further analyzing its effects on time and cost of the whole product development process.

## 3. Modeling Design Iteration Based on Tearing Approach and Inner Iteration Technology

### 3.1. The Limitations of Classic WTM Model for Identifying Design Iteration

**Figure 1:**
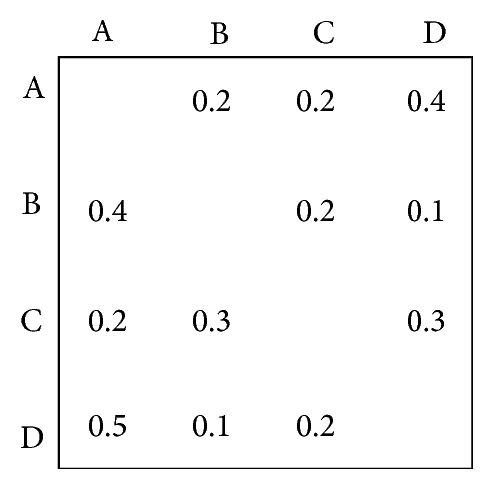
The sample of a WTM model.

In the classic WTM model, the entries either in every row or in every column of WTM sum to less than one so as to assure that doing one unit of work in some task during an iteration will create less than one unit of work for that task at a future stage. Such design and development process will converge. However, in real-world product design and development process, some unexpected situations may occur. For example, there is no technically feasible solution to the given specifications or the designers are not willing to compromise to reach a solution, which represents that the corresponding design process will not converge and the entries either in every row or in every column of WTM sum to more than one.  Figure 1 denotes this situation. As can be seen from it the entries in the first column sum to 1.1  (i.e.,  0.4 + 0.2 + 0.5 = 1.1). This design and development process is unstable and the whole process will not converge.

**Figure 2:**
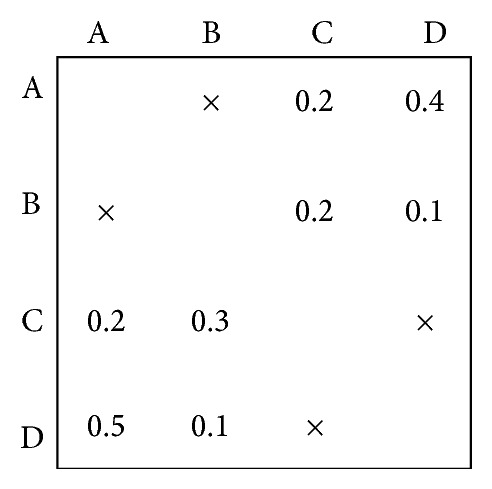
The sample of a WTM model after tearing approach.

Tearing is the process of choosing the set of feedback marks that if removed from the matrix (and then the matrix is repartitioned) will render the matrix a lower triangular one. The marks that we remove from the matrix are called “tears” [23]. According to its definition, an original large coupled set can be transformed into some small ones through tearing approach. In doing so, these small coupled sets may easily satisfy precondition of WTM. Take the coupled set shown in Figure 1 as an example; after tearing approach, two small ones (i.e., (A, B) and (C, D)) are obtained as shown in Figure 2. We can see from Figure 2 that the entries either in every row or in every column of these two coupled sets sum to less than one and WTM model can be used in this situation.

**Figure 3:**
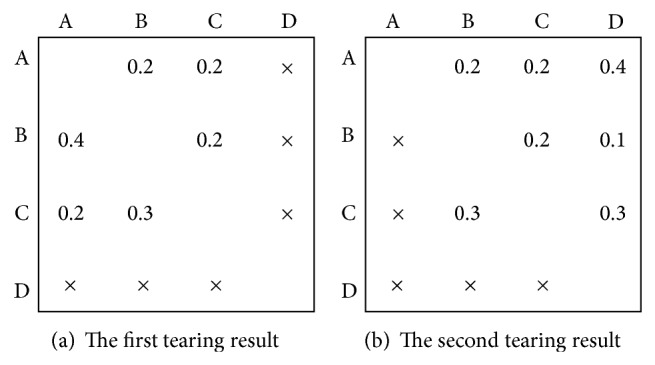
Different results after tearing approach.

However, because tearing algorithm neglects dependencies among tasks in fact, some quality losses may be generated. Therefore, how to reduce these quality losses needs to be studied. In Figure 2, there exist many tearing results. For instance, Figure 3 shows two different results using tearing approach and diverse quality losses can be obtained, where the symbol “×” denotes dependencies neglected among tasks.

According to the analysis mentioned above, it is easy to find that the tearing approach can transform the large coupled set into some small ones but may bring some quality loss. As a result, quality loss is one of the important indexes when using tearing approach to deal with coupled sets. In addition, development cost is another important index that should be considered when using WTM model. In this paper, a hybrid iteration model used to solve coupled sets is set up. In this model, two objectives including quality loss and development cost are defined and the constrained condition is proposed so as to satisfy the premise of WTM model. The following section will go on analyzing how to build this model.

### 3.2. Modeling Design Iteration Based on Hybrid Iteration Strategy

For a coupled set *C*, its execution time TT (total time) includes consuming time of task transmission and interaction. Define the task execution sequence after tearing as *L* and the abstract model of this problem is

(1) $$\begin{array}{c} \min\text{TT}=\theta \left(L\right), \end{array}$$

where the target of tearing operator is to search for a feasible task execution sequence so as to make execution time shortest; however, formula (1) is very abstract and needs further discussion. *L* denotes a feasible task execution sequence after tearing a coupled set. Every feasible task sequence corresponds to a kind of time consumption. The relationship is expressed:

(2) $$\begin{array}{c} T_{i}=\varphi \left(L_{i}\right), \end{array}$$

where *L*_*i*_ represents task sequence through the *i*th tearing operation for a coupled task set and function *φ*() is used to calculate the corresponding design and execution consuming time of *L*_*i*_ task sequence.

Suppose the coupled task set has *n* kind of way for tearing; combining with formula (2), formula (1) can be transformed into

(3) $$\begin{array}{c} \min\text{TT}=\min\left\{T_{1},T_{2},\ldots ,T_{n}\right]. \end{array}$$

Formula (3) is time aggregative model based on task transmission and interaction. As can be seen from this model the shortest task transmission and interaction represent an optimal task execution sequence. According to this task sequence, the whole design duration of coupled set will come to the shortest one. Moreover, the measurement of aggregative time is to calculate the execution time *T*_*i*_ of all the tasks. The measurement of task transmission and interaction is described as follows:

(4) $$\begin{array}{c} t_{r}=SF\times t, \end{array}$$

where *t*_*r*_ is practical transmission time. *SF* can be calculated by the following formula, where *m* is the number of impact influences, *V*_*i*_ is the value of *F*_*i*_, and *e*_*i*_ is the weight of *F*_*i*_:

(5) $$\begin{array}{c} SF=\overset{m}{\underset{i=1}{\sum }}e_{i}\times V_{i}. \end{array}$$

According to the analysis, the model can be built based on the following assumptions [18].

1. All tasks are done in every stage.
2. Rework performed is a function of the work done in the previous iteration stage.
3. The work transformation parameters in the matrix do not vary with time.

We take formula (5) mentioned above as the first objective function which is used to measure the quality loss of decoupling process. The other objective function, development cost, is adopted by using cumulative sum of the whole iteration process. In addition, the constraint condition of the model can be expressed as follows: *Ω*_*j*_ = ∑_*i*=1_^*n*^ *a*_*ij*_ < 1  (*i*, *j* ∈ *A*_*k*_), which makes the entries either in every row or in every column sum to less than one. Based on these analyses, the hybrid model set up in this paper is described as follows:

(6)  Object 1:  tr=SF×t,

(7)  Object 2:lim⁡T→∞⁡∑t=0TΛt=I−Λ−1,

(8)  Satisfy Ωj=∑i=1naij<1 i,j∈Ak,

where formulas (6) and (7) are objective functions, where the first one represents quality loss and the other development cost. The symbol *A*_*k*_ in constraint condition (8) denotes small coupled sets after tearing approach and *a*_*ij*_ is an element in *A*_*k*_. This constraint condition is used to assure that the decomposed small coupled set *A*_*k*_ can converge.

## 4. Artificial Bee Colony Algorithm for Finding a Near-Optimal Solution

The hybrid model set up in the above section is difficult in finding out the optimal solution by conventional methods such as branch and bound method and Lagrangian relaxation method. Due to its simplicity and high-performance searching ability, heuristic algorithm has been widely used in NP-hard problems. As a new swarm intelligence algorithm, artificial bee colony algorithm (ABC) has strong local and global searching abilities and has been applied to all kinds of engineering optimization problems. In this section, the ABC algorithm is used to solve this coupled problem.

### 4.1. Artificial Bee Colony Algorithm

The ABC algorithm is one of the most recently introduced optimization algorithms inspired by intelligent foraging behavior of a honey bee swarm. It was firstly proposed by Karaboga [24] for optimizing multivariable numerical functions. Furthermore, Basturk et al. [25] also applied ABC to function optimizations with constraints and the simulation results had shown that this intelligent algorithm is superior to other heuristic algorithms such as ant colony optimization (ACO) [26], particle swarm optimization (PSO) [27], and artificial plant optimization (APO) [28] in 2006. In addition, the ABC algorithm has been also used to solve large-scale problems and engineering design optimization. Some representative applications are introduced as follows. Singh [29] applied the ABC algorithm for the leaf-constrained minimum spanning tree (LCMST) problem and compared the approach against GA, ACO, and tabu search. In literature [29], it was reported that the proposed algorithm was superior to the other methods in terms of solution qualities and computational time. Zhang et al. [30] developed the ABC clustering algorithm to optimally partition *N* objectives into *K* cluster and Deb's rules were used to direct the search direction of each candidate. Pan et al. [31] used the discrete ABC algorithm to solve the lot-streaming flow shop scheduling problem with the criterion of total weighted earliness and tardiness penalties under both the idling and no-idling cases. Samanta and Chakraborty [32] employed ABC algorithm to search out the optimal combinations of different operating parameters for three widely used nontraditional machining (NTM) processes, that is, electrochemical machining, electrochemical discharge machining, and electrochemical micromachining processes. Chen and Ju [33] used the improved ABC algorithm to solve the supply chain network design under disruption scenarios. The computational simulations revealed the ABC approach is better than others for solving this problem. Bai [34] developed wavelet neural network (WNN) combined with a novel artificial bee colony for the gold price forecasting issue. Experimental results confirmed that the new algorithm converged faster than the conventional ABC when tested on some classical benchmark functions and was effective in improving modeling capacity of WNN regarding the gold price forecasting scheme. All these researches illustrated that the ABC algorithm has powerful ability to solve much more complex engineering problems [35, 36].

In the basic ABC algorithm, the colony of artificial bees contains three groups of bees: employed bees, onlookers, and scouts. Employed bees determine a food source within the neighborhood of the food source in their memory and share their information with onlookers within the hive, while onlookers select one of the food sources according to this information. In addition, a bee carrying out random search is called a scout. In ABC algorithm, the first half of the colony consists of the employed bees and the remaining half includes the onlookers. There is only one employed bee corresponding to one food source. That is to say, the number of employed bees is equal to the number of food sources around the hive. The position of a food source denotes a possible solution for the optimization problem and the nectar amount of a food source corresponds to the quality (fitness) of the associated solution.

The initial population of solutions is filled with *SN* number of randomly generated *D*-dimensional real-valued vectors (i.e., food sources). Each food source is generated as follows:

(9) $$\begin{array}{c} x_{i}^{j}=x_{\min}^{j}+\text{rand}\left(0,1\right)\left(x_{\max}^{j}-x_{\min}^{j}\right), \end{array}$$

where *i* = 1,2,…, *SN*, *j* = 1, 2,…, *D*, and *x*_min⁡_^*j*^ and *x*_max⁡_^*j*^ are the lower and upper bounds for the dimension *j*, respectively. These food sources are randomly assigned to *SN* number of employed bees and their fitness is evaluated.

In order to produce a candidate food position from the old one, the ABC used the following equation:

(10) $$\begin{array}{c} v_{ij}=x_{ij}-\varphi_{ij}\left(x_{ij}-x_{kj}\right), \end{array}$$

where *j* ∈ {1,2,…, *D*} and *k* ∈ {1,2,…, *SN*} are randomly chosen indexes. Although *k* is determined randomly, it has to be different from *i*. *φ*_*ij*_ is a random number in the range [−1, 1]. Once *V*_*i*_ is obtained, it will be evaluated and compared to *X*_*i*_. If the fitness of *V*_*i*_ is equal to or better than that of *X*_*i*_, *V*_*i*_ will replace *X*_*i*_ and become a new member of the population; otherwise *X*_*i*_ is retained.

After all employed bees complete their searches, onlookers evaluate the nectar information taken from all employed bees and choose one of the food source sites with probabilities related to its nectar amount. In basic ABC, roulette wheel selection scheme in which each slice is proportional in size to the fitness value is employed as follows:

(11) $$\begin{array}{c} P_{i}=\frac{\text{fit}\left(x_{i}\right)}{\sum_{n=1}^{SN}\text{fit}\left(x_{n}\right)}, \end{array}$$

where fit(*x*_*i*_) is the fitness value of solution *i*. Obviously, the higher the fit(*x*_*i*_) is, the more the probability is that the *i*th food source is selected.

If a position cannot be improved further through a predetermined number of cycles, then that food source is assumed to be abandoned. The scouts can accidentally discover rich, entirely unknown food sources according to (9). The value of predetermined number of cycles is called “*limit*” for abandoning a food source, which is an important control parameter of ABC algorithm.

**Figure 4:**
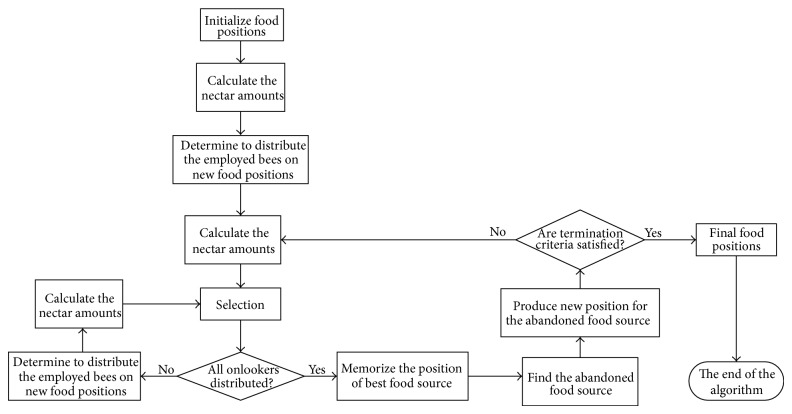
The flowchart of the artificial bee colony algorithm.

There are three control parameters used in the basic ABC: the number of the food sources which is equal to the number of employed bees (*SN*), the value of* limit*, and the maximum cycle number (*MEN*).  Figure 4 summarizes the steps of the basic ABC.

### 4.2. A Novel Artificial Bee Colony Algorithm for Identity Design Iteration

**Figure 5:**
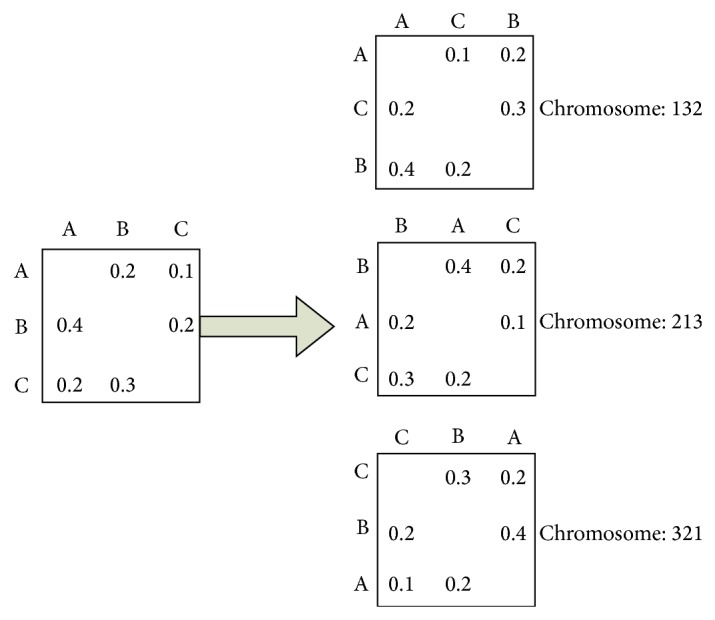
The sample of encoding process.

**Figure 6:**
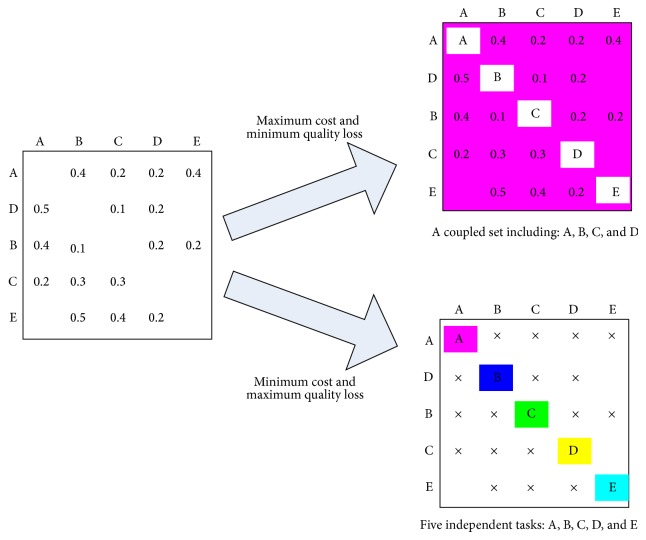
Two extreme cases of coupled set decomposition.

**Figure 7:**
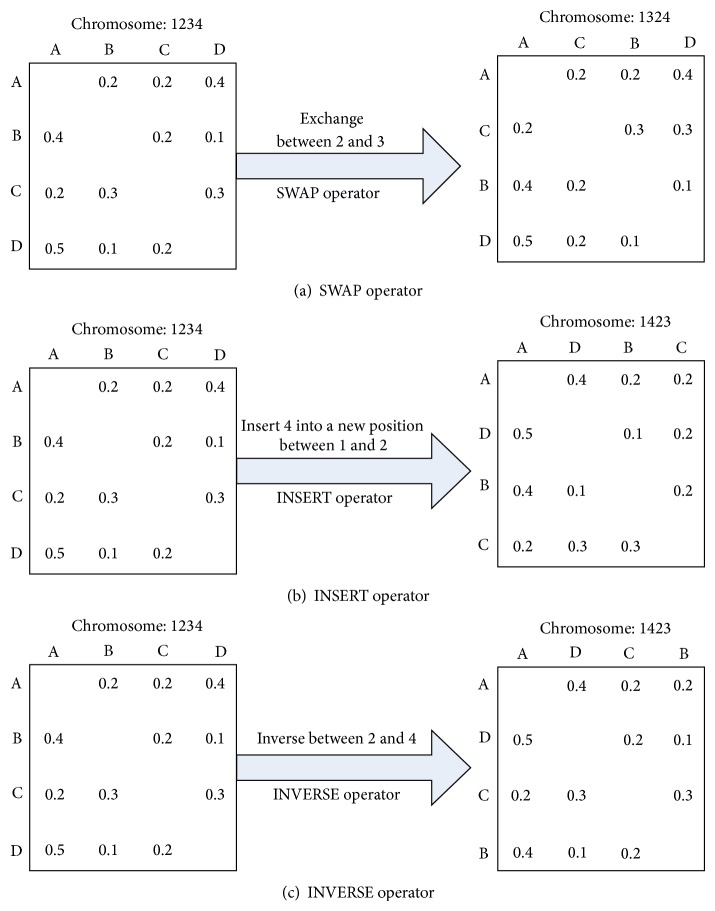
Generation of neighborhood solution.

The iteration model built in Section 3 is a typical NP-hard problem. Therefore, it is difficult to find out the optimal solution using conventional technologies. In the past decades, ABC algorithm, as a typical method of swarm intelligence, is more suitable to solve combination optimization problems. However, the basic ABC algorithm mentioned in Section 4.1 is only designed to solve continuous function optimization problems and is not suitable for discrete problems. As a result, in this section, we design discrete ABC algorithm to solve coupled sets and the detailed process is shown as follows.

(*1) Solution Representation*. According to the characteristics of the problem, real number encoding is adopted. The solution representation is shown in Figure 5. Because matrix *A* = (*a*_*ij*_)_*n*×*n*_ includes three rows and three columns, the real numbers 1, 2, and 3 represent the corresponding row and column of DSM matrix, respectively. Figure 5 shows three different chromosomes representing three different spread patterns.

(*2) Population Initialization*. To guarantee an initial population with certain quality and diversity, we use two strategies. One is to assign a randomly generated solution to every employed bee; the other is to generate a portion of food sources by using experiential knowledge so as to describe the uncoupled schemes having less quality loss or lower development cost. 

 (*3) Food Source Evaluation*. In this discrete ABC algorithm, there are two indexes used to evaluate food source: one is the quality loss when using tearing approach described by formula (6); the other is development cost caused by iteration process and it is defined by formula (7). Note that these two objectives are mutually exclusive. It means the more the quality losses are the lower the development cost is and vice versa. The two extreme cases are corresponding to the maximum quality loss and the minimum development cost shown in Figure 6. As can be seen from Figure 6 suppose that the coupled set is composed of 5 tasks. In the first situation, if tearing approach is not used, there exists no quality loss in development process and WTM model is used to analyze the coupled set. However, the entries either in every row or in every column should sum to more than one so as to satisfy the premise of WTM model. Otherwise, the whole development process does not converge. The other situation represents that the dependencies among tasks are not considered and the large coupled set is decomposed into five independent tasks. The development cost is equal to the sum of these five tasks' cost which is described by execution time of tasks. In this situation, due to no iterations existing, the development cost is the minimum. The target of the ABC algorithm is to search a feasible decoupling scheme in order to reduce development cost and quality loss as well. In this paper, setting weights are adopted to transform a multiple-objective problem into a single-objective one so as to simplify problem-solving process. 

(*4) Employed Bee Phase.* The employed bees generate food sources in the neighborhood of their position in the ABC algorithm. In this paper, three operations including SWAP, INSERT, and INVERSE are used to produce neighboring solutions, where the SWAP operator is defined by interchanging two tasks in different positions, while the INSERT one is defined by removing a task from its original position and inserts it into a new position and the last one, INVERSE. generates a neighbor by inversing the sequence between two tasks in different positions. The detailed representation is shown in Figure 7. Note that if the neighboring solutions do not satisfy preference constraints, the old one should be retained. Furthermore, in order to enrich searching region and diversify the population, five related approaches based on SWAP, INSERT, or INVERSE operators are adopted to produce neighboring solutions, which are shown as follows:

1. performing one SWAP operator to a sequence;
2. performing one INSERT operator to a sequence;
3. performing two SWAP operators to a sequence;
4. performing two INSERT operators to a sequence;
5. performing two INVERSE operators to a sequence.

The food sources in the neighborhood of their position mentioned above may have different performances in evaluation process, so a feasible self-learning form should be selected. In addition, for the selection of food sources, if new food source is better than the current one, the new one should be accepted. It also means the greedy selection is adopted. 

(*5) Onlooker Bee Phase*. In the basic ABC algorithm, an onlooker bee chooses a food source depending on the probability value associated with that food source. In other words, the onlooker bee chooses one of the food sources after making a comparison among the food sources around current position, which is similar to “roulette wheel selection” in GA. In this paper, we also retain this approach to make the algorithm converge fast.

(*6) Scout Bee Phase*. In the basic ABC algorithm, a scout produces a food source randomly. This will decrease the search efficacy, since the best food source in the population often carried better information than others. As a result, in this paper, the scout produces a food source using several SWAP, INSERT, and INVERSE operators to the best food source in the population. In addition, to avoid the algorithm trap into a local optimum, this process should be repeated several times. 

(*7) Disposal of Constraint Condition*. The constraint condition may affect the feasibility of decoupling scheme. As a result, we introduce penalty function method to dispose of constraint condition and make the scheme that does not satisfy constraint condition have a lower possibility to be selected in the next generation.

## 5. Application Example

In this section, a numerical example deriving from an engineering design of a chemical processing system [37] is utilized so as to help to understand the proposed approach. In this example, an engineering design of a chemical processing system has 20 tasks and detailed task information is listed in Table 1. Firstly, DSM method is used to model the dependencies among tasks; then analytic hierarchy process (AHP) is adopted to set up 0-1 DSM and partitioning algorithm is used to find out the coupled sets existing in DSM; subsequently, the hybrid iteration model proposed in this paper is introduced to deal with the decoupling problem; finally, the simulation is obtained.

**Figure 8:**
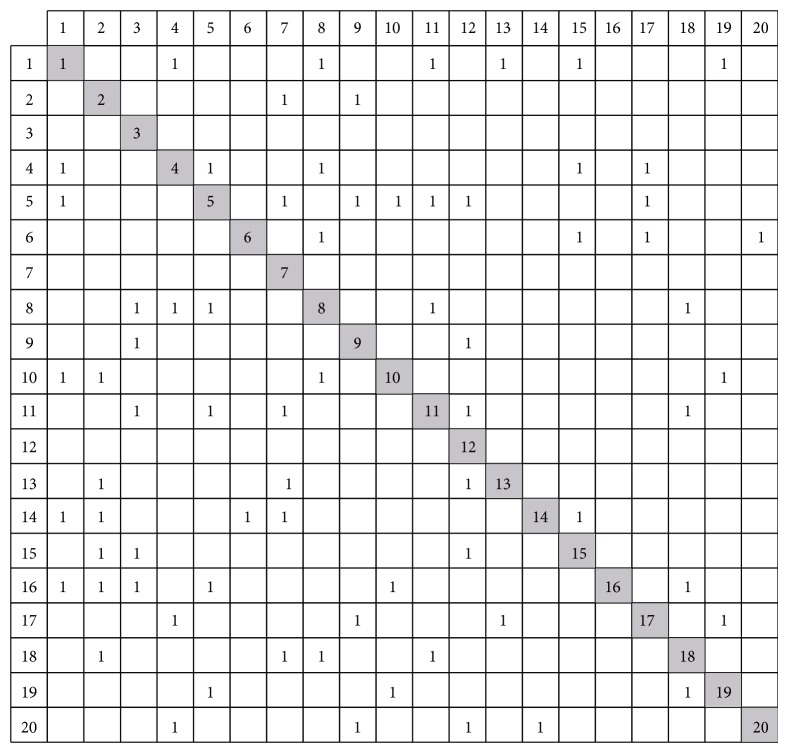
Boolean DSM matrix.

In the first step, according to dependency modeling technology mentioned in literature [2], the DSM model is set up as shown in Figure 8, where the empty elements represent no relationships between two tasks and number “1” represents input or output information among tasks. For example, task 1 requires information from tasks 13 and 15 when it executes. Additionally, task 1 must provide information to tasks 4, 5, 10, 14, 16, and 18; otherwise they cannot start. Nevertheless, Figure 8 only denotes the “existence” attributes of a dependency between the different tasks. In order to further reveal their matrix structure, it is necessary to quantify dependencies among tasks.

**Figure 9:**
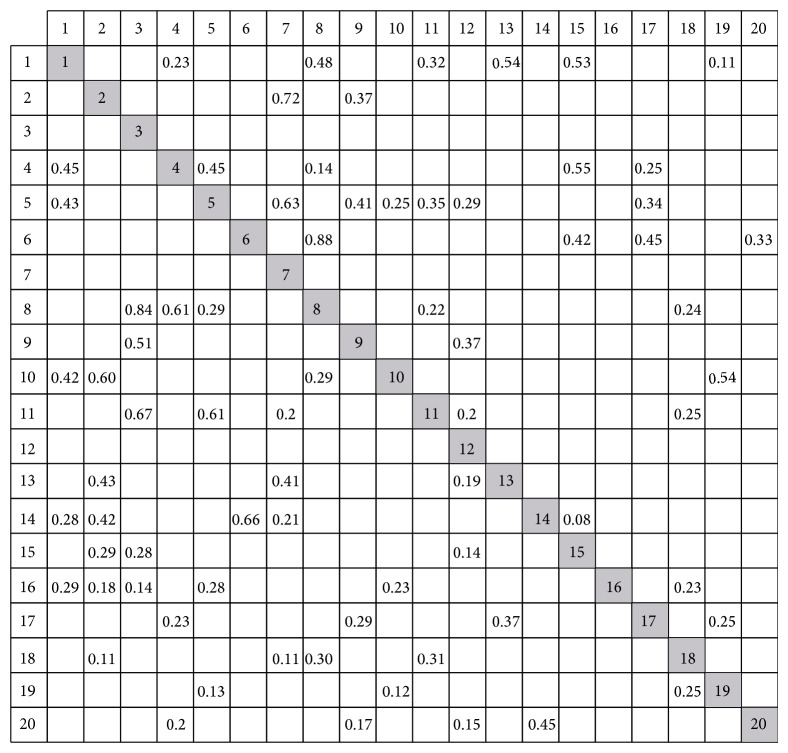
Numerical DSM matrix.

Because quantification of dependencies among tasks is helpful to reveal essential features of tasks, we introduce a two-way comparison scheme [4] to transform the binary DSM into the numerical one. The main criteria of this approach are to perform pairwise comparisons in one way for tasks in row and in another way for tasks in columns to measure the dependency between different tasks. In the row-wise perspective, each task in rows will serve as a criterion to evaluate the relative connection measures for the nonzero elements in that row. It means that for each pair of tasks in rows, which one can provide more input information than the other. Similarly, in the column-wise perspective, each task in columns will serve as a criterion to evaluate the relative connection measures in that column. It also means that for every pair of tasks compared in columns, which one can receive more output information than the other. The detailed process is omitted due to the length limitation of this paper and authors may refer to literature [4] to know of this approach. The final numerical DSM is shown in Figure 9.

**Figure 10:**
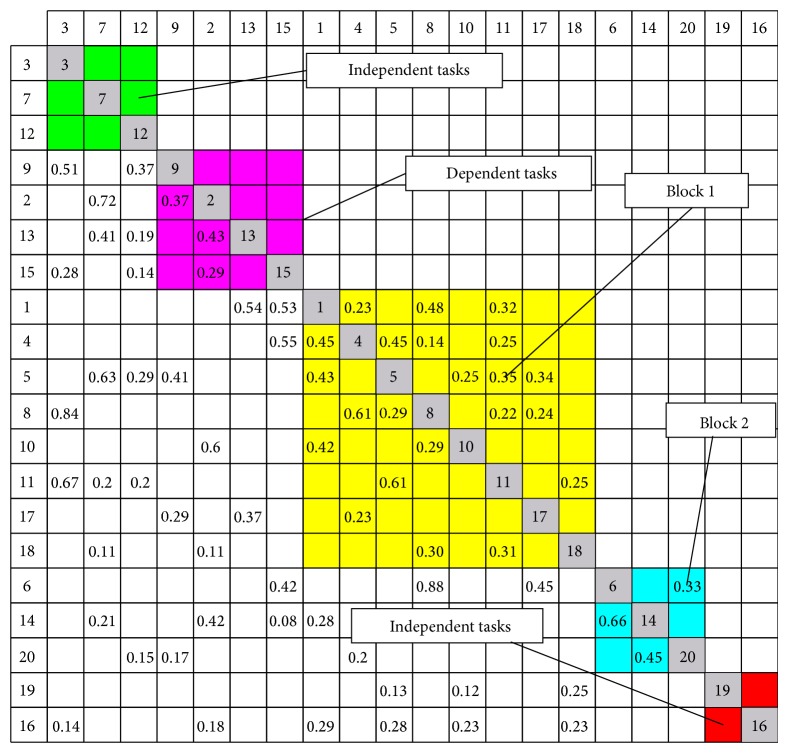
Results after partitioning algorithm.

Subsequently, partitioning algorithm is adopted and five subprocesses have been obtained as shown in Figure 10. The first subprocess contains 3 tasks such as 3, 7, and 12, and all of them can be executed without input information from others; the second one consists of tasks 2, 9, 13, and 15, and they must receive information from the first subprocess; the third one is a large coupled set including tasks 1, 4, 5, 8, 10, 11, 17, and 18, and all the tasks are interdependent; the fourth one is a small coupled set comprised of tasks 6, 14, 16, 19, and 20, where all the tasks must depend on information from the first, the second, and the fourth subprocess. The fifth one includes tasks 16 and 19 and all the tasks are independent. As can be seen from Figure 10 block 2 is a small coupled set and the classic WTM can be used to solve this problem. However, block 1 is a large coupled set and the entries either in every row or in every column of WTM sum to more than one, so the hybrid iteration method should be used in this situation.

**Figure 11:**
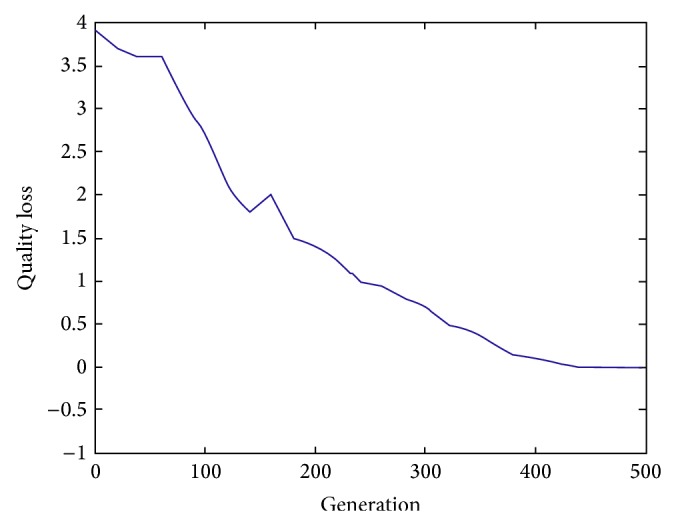
The change curve of quality loss.

**Figure 12:**
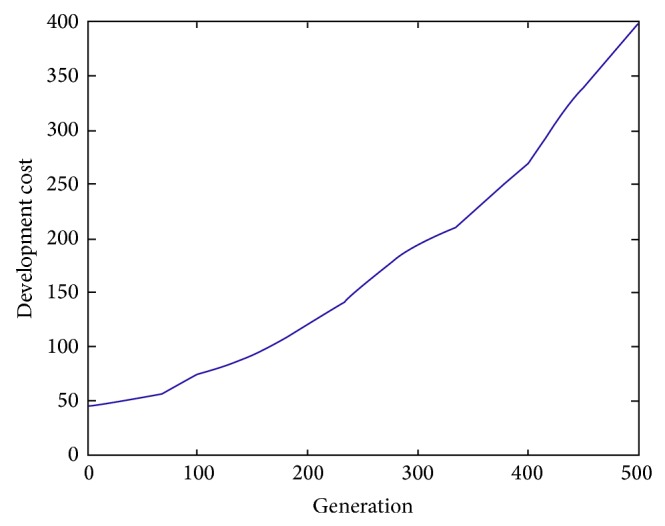
The change curve of development cost.

When using the hybrid iteration model, tearing approach is applied to transform the large coupled set into some small ones and then improved ABC algorithm is used to find the optimal decoupling schemes according to measuring two objectives including quality loss and development cost as well. The related parameters of ABC algorithm are set as follows: *SN* = 10, *limit* = 20, and *MEN* = 500. The simulations results are shown in Figures 11 and 12. Due to the exclusiveness of these two objectives, the best tearing result should bring the minimum quality loss and the original coupled set does not decompose. Nevertheless, the iteration process does not converge and the development process is not feasible. In addition, the minimum development cost corresponds to eight independent tasks and all relationships among tasks are not considered. The development cost can be calculated as follows: 6 + 8 + 4 + 3 + 5 + 9 + 5 + 5 = 45 (Yuan/Time).

**Figure 13:**
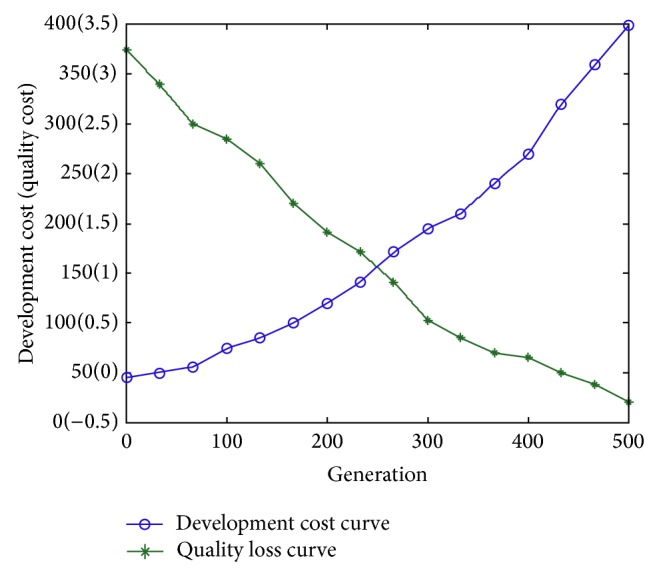
The change curve of objective function.

Furthermore, the effects of the double-objectives on the coupled set decomposition are analyzed.  Figure 13 describes the change curves including these two objectives. We can see from it that different schemes have their own advantages. Decision makers can select different design iteration process according to practical product development requirements. For example, Table 2 displays development cost and quality loss corresponding to different decoupling schemes and design engineer can choose different strategies to decompose large coupled sets. According to different strategies, expected objectives may be achieved at the expense of the other ones. All in all, the higher the development cost is, the lower the quality loss is and vice versa.

## 6. Conclusions

In this paper, the shortcomings existing in WTM model are discussed and tearing approach as well as inner iteration method is used to complement the classic WTM model. In addition, the ABC algorithm is also introduced to find out the optimal decoupling schemes. The main works are as follows: firstly, tearing approach and inner iteration method are analyzed for solving coupled sets; secondly, a hybrid iteration model combining these two technologies is set up; thirdly, a high-performance swarm intelligence algorithm, artificial bee colony, is adopted to realize problem-solving; finally, an engineering design of a chemical processing system is given in order to verify its reasonability and effectiveness.

The future research may focus on how to extend the model to other real-world practices. In addition, how to further improve the performance of the ABC algorithm is another issue needing to be studied.

## Figures and Tables

**Table 1:** Task information for an engineering design of a chemical processing system [33].

Number	Description of tasks	Duration (day)	Predecessor
1	Operating structure design	6	13, 15
2	Vessel design	12	7, 9
3	Plant layout/general arrangement	5	
4	Shipping design	8	1, 15
5	Structure lifting design	4	1, 7, 9, 12
6	Pressure drop analysis	4	8, 15, 17
7	Process engineering	3	
8	Structural documentation	3	3, 4, 5
9	Size valves	4	3, 12
10	Wind load design	5	1, 2, 8
11	Seismic design	9	3, 5, 7, 12
12	Piping design	4	
13	Process and instrumentation diagram	2	2, 7, 12
14	Equipment support	2	1, 2, 6, 7, 15
15	Pipe flexibility analysis	2	2, 3, 12
16	Design documentation	4	1, 2, 3, 5, 10, 18
17	Foundation load design	5	4, 9, 13
18	Insulation structural design	5	1, 2, 7, 8, 11
19	Structural bill of material (BOM)	6	5, 10, 18
20	Assembly design	7	4, 9, 12, 14

**Table 2:** Decoupling schemes of the coupled set.

Number	Decomposition scheme	Quality loss	Development cost	Feature
1	Eight independent tasks	*∞*	45	Minimum cost and maximum quality cost
2	Coupled sets: (5, 10, 11) and (1, 4, 8, 10, 18)	0.32	361.5	Maximum cost and minimum quality cost
3	Coupled sets: (1, 4, 5, 8) and (10, 11, 17, 18)	1.71	236.1	Medium cost and quality loss
4	Coupled sets: (1, 5, 10, 17) and (4, 8, 11, 17)	2.59	297.6	Alternative solutions
